# Stable transfection system for *Babesia* sp. Xinjiang

**DOI:** 10.1186/s13071-021-04940-x

**Published:** 2021-09-09

**Authors:** Jinming Wang, Xiaoxing Wang, Guiquan Guan, Jifei Yang, Junlong Liu, Aihong Liu, Youquan Li, Jianxun Luo, Hong Yin

**Affiliations:** 1 State Key Laboratory of Veterinary Etiological Biology, Key Laboratory of Veterinary Parasitology of Gansu Province, Lanzhou Veterinary Research Institute, Chinese Academy of Agricultural Science, Xujiaping 1, Lanzhou, 730046 Gansu China; 2 Jiangsu Co-Innovation Center for the Prevention and Control of Important Animal Infectious Disease and Zoonose, Yangzhou University, Yangzhou, 225009 China

**Keywords:** *Babesia* sp. Xinjiang, Stable transfection, Genetic manipulation, Cross-over homologous recombination

## Abstract

**Background:**

Stable transfection systems have been described in many protozoan parasites, including *Plasmodium falciparum*, *Cryptosporidium parvum*, *Babesia bovis*, *Babesia ovata*, and *Babesia gibsoni*. For *Babesia* sp. Xinjiang (*Bxj*), which is the causative pathogen of ovine babesiosis and mainly prevails across China, the platform of those techniques remains absent. Genetic manipulation techniques are powerful tools to enhance our knowledge on parasite biology, which may provide potential drug targets and diagnostic markers.

**Methods:**

We evaluated the inhibition efficiency of blasticidin (BSD) and WR99210 to *Bxj*. Then, a plasmid was constructed bearing selectable marker BSD, green fluorescent protein (GFP) gene, and rhoptry-associated protein-1 3′ terminator region (*rap* 3′ TR). The plasmid was integrated into the elongation factor-1 alpha (*ef-1α*) site of *Bxj* genome by cross-over homologous recombination technique*.* Twenty μg of plasmid was transfected into *Bxj* merozoites. Subsequently, drug selection was performed 24 h after transfection to generate transfected parasites.

**Results:**

Transfected parasite lines, *Bxj*-c1, *Bxj*-c2, and *Bxj*-c3, were successfully obtained after transfection, drug selection, and colonization. Exogenous genes were integrated into the *Bxj* genome, which were confirmed by PCR amplification and sequencing. In addition, results of western blot (WB) and indirect immunofluorescence assay (IFA) revealed that GFP-BSD had expressed for 11 months.

**Conclusions:**

In our present study, stable transfection system for *Bxj* was successfully developed. We anticipate that this platform will greatly facilitate basic research of *Bxj*.

**Graphical abstract:**

**Figure:**
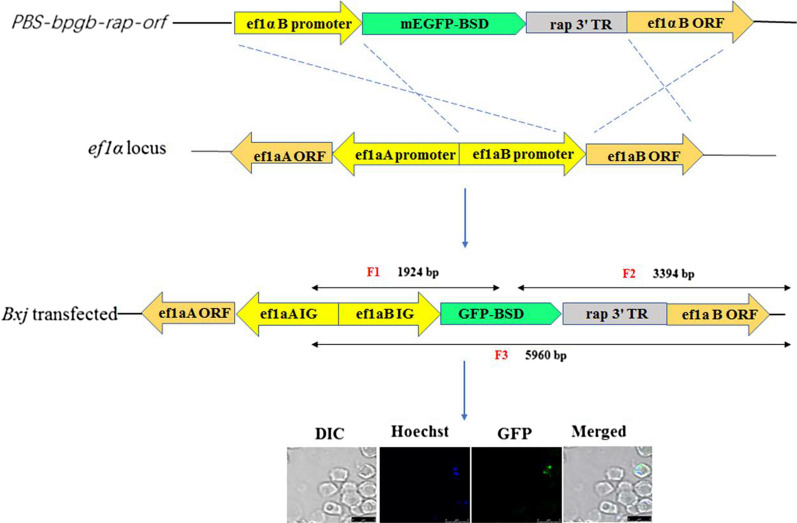

## Background

Babesiosis, caused by protozoan pathogens of the genus *Babesia* (phylum Apicomplexa, order *Piroplasmida*) infective to humans and domestic and wild animals, is one of the emerging and re-emerging tick-borne disease in tropical and subtropical regions of the world [1, 2]. A wide spectrum of clinical manifestation ranges from mild fever to serve anemia hemoglobinuria and even death [3]. Early in the nineteenth century, the first case of babesiosis was reported in Rumania and correlated with bovine hemoglobinuria or red water fever. Shortly after, similar organisms were also determined in sheep red blood cells [4]. Till now, more than 100 *Babesia* species have been described throughout the world. However, only a few *Babesia* species have been identified in sheep and cause ovine babesiosis, namely *B. ovis*, *B. motasi*, and *B. crassa*.

In China, ovine babesiosis was firstly reported as early as 1982 in Sichuan Province and 1986 in Heilongjiang Province [5, 6]. Since then, this disease was sporadically reported in Xinjiang Uygur Autonomous region, Henan, Shannxi, Yunnan, and Hebei Province. During the past decades of investigations of ovine babesiosis, great attention had been paid to a novel *Babesia* species, *Babesia* sp. Xinjiang, which presented distinct morphologies from *B. motasi*, *B. ovis*, and *B. crassa* in a thin blood smear [7, 8]. This novel *Babesia* species was initially isolated from a splenectomized sheep infested with partially engorged *Rhipicephalus sanguineus* and *Hyalomma anatolicum anatolicum* ticks [9]*.* In the years since then, systematic studies of this *Babesia* species have investigated morphological characteristics, transmission patterns, culture features, epidemiology, pathogenicity, and whole-genome sequencing and annotation [10–14]. Available results indicated that this pathogen is widely distributed across China and has caused significantly economic losses to the sheep industry [15].

During recent decades, much attention has been focused on developing diagnostic assays and performing epidemiological investigations. However, available approaches to evaluate virulence factors and vaccine candidate antigens, and even to explore invasion and transmission mechanisms for these parasites, are limited. Ovine babesiosis control suffers from a lack of effective vaccines and limited choices of therapeutic drugs. Development of these relies on a better understanding of the basic biology of *Babesia* species [16]. Genetic manipulation technologies have been described to discover virulence factors and to investigate the interaction of parasite and host cells in apicomplexan parasites such as *Cryptosporidium parvum*, *Babesia bovis*, *B. gibsoni*, *B. ovis*, *B. ovata*, *Theileria annulata*, and *T. parva* [17–25]. In this study, we developed a stable transfection system of *Bxj* merozoites to investigate tick–*Babesia* and host–*Babesia* interactions in the future.

## Methods

### In vitro culture system

*Bxj* was cultured in 24-well plates (Corning, MA, USA) at 37 °C under an atmosphere of 5% CO_2_ as reported previously [14, 15, 26]. Briefly, the parasite was cultured in 7.5% fresh sheep erythrocytes supplemented with 20% fetal bovine serum (FBS) (Gibco, Carlsbad, CA, USA) in Roswell Park Memorial Institute 1640 medium (Lonza Biologics, Portsmouth, NH, USA).

### Evaluation the inhibition efficiency of BSD and WR99210 to *Bxj*

*Bxj* was cultured in complete medium with various concentrations of BSD (1 μg/ml, 2 μg/ml, 5 μg/ml, 8 μg/ml, and 10 μg/ml) and WR99210 (10 μg, 25 μg/ml, 50 μg/ml, 100 μg/ml, and 200 μg/ml), and the medium was replaced each day. The proportion of infected red blood cells (PI) was calculated by examining 2000 red blood cells (RBCs) stained with Giemsa in thin blood smear at 48 h [22]. The formula of *Bxj* growth inhibition was as follows:

$$\left( {{\text{mean}}\;{\text{PI}}\;{\text{in control wells}}\,\,{-}\,\,{\text{mean PI in wells containing BSD}}} \right)/{\text{mean PI in control wells}}\, \times \,{1}00\%$$

### Plasmid constructs

**Fig. 1:**
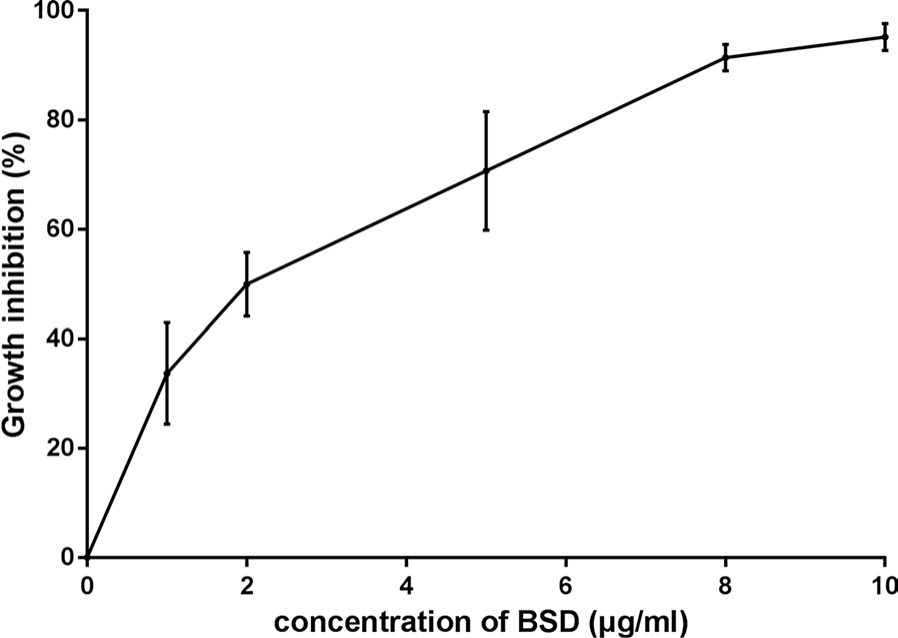
*Babesia* sp. Xinjiang sensitivity to BSD. All data are presented as means ± SD of triplicate cultures

The plasmid construct used in this study is listed as Fig. 1 (*PBS-bpgb-rap-orf*). Initially, the *ef1α-B* 5′ non-coding region and enhanced green fluorescent protein (eGFP) gene and blasticidin-S (BSD) deaminase gene were amplified from *Bxj* genomic DNA and plasmid *pgfp-bsd-ef* (kindly donated by Carlos E. Suarez) using the primer sets listed in Table 1. Then, these two sequences were cloned into the NotI site of plasmid pBluescript SK(+) using a ClonExpress MultiS One Step Cloning Kit (Vzayme, Nanjing, China) according to the manufacturer’s instructions. This plasmid was designated as *PBS-bpgb*. Meanwhile, *Bxj rap*3′ terminal region (*Bxj rap*3′ TR) and *ef1α–B* open reading frame (*ef1α–B-orf*) sequences were amplified from *Bxj* genomic DNA and cloned into the NotI site of plasmid pBluescript SK(+) as mentioned above, and the generated plasmid was designated as *PBS-TR-orf.* Subsequently, the first large fragment of the *ef1a-B* non-coding region and *gfp-bsd* was amplified from plasmid *PBS-bpgb*, and the second fragment of the *Bxj rap*3' TR and *ef1α–B* open reading frame sequences was derived from plasmid *PBS-TR-ORF.* Finally, these two large fragments were ligated into the NotI site of plasmid pBluescript SK(+), designated as *PBS-bpgb-rap-orf*. Furthermore, cloned plasmids were validated by polymerase chain reaction (PCR) amplification using two sets of primers (set 1: *ef1a-B-F* and *ef1a-B-R*; set 2: *ef1a-orf-F* and *ef1a-orf-R*) and confirmed by sequencing and extracted using a QIAGEN Plasmid Maxi Kit (Qiagen, Hilden, Germany) according to the manufacturer’s instructions.

**Table 1:** Information of primer sets used in this study

Gene	Sequence (5′-3′)	Size of target sequence	Amplified fragment
*ef1a-B-F*	ACTAGTTCTAGAGCGGCCGCGTTGTCTTTTGGTATTAC	958	*ef1a-B* promoter sequence
*ef1a-B-R*	CGCCCTTGCTCACCATTCTAGATTTCGTGGAGTTTTAACT		
*gfp-bsd-F*	ATGGTGAGCAAGGGCGAGGAG	1113	*gfp-bad* gene
*gfp-bsd-R*	AGCTCCACCGCGGTGGCGGCCGCTTAGCCCTCCCACACATAAC		
*rap-F*	GGGAGGGCTAAGCGGCCGCGGCAGCTTCAAAGCG	1362	*rap*3' TR
*rap-R*	GGGTCTTCTCCTTCGGCATTCTTAACACCAGGCAAT		
*ef1a-orf-F*	ATGCCGAAGGAGAAGACCC	1347	*ef1α-B orf*
*ef2a-orf-R*	CCGCGGTGGCGGCCGCTCACTTCTTGGCGGCCTTCTGGGC		
*efbs-F*	GAGCGTCGAATTATTAACTGGG	1924	F1
*efbs-R*	CTTGTACAGCTCGTCCATGC		
*gfor-F*	GCCAAGCCTTTGTCTCAAGAAG	3394	F2
*gfor-R*	CTAGCAGTGTGTTAGCGTGC		
*orf-R*	GCACGATTCCATCGCTACAACG	5960	F3 (primer set *efbs-F* and *orf-R*)

Homologous sequences are underlined and are employed to connect with upstream and downstream genes

### Electroporation of *Bxj* merozoites and drug selection

When infected RBCs reached 10–20%, the cultures in a 75 cm^2^ flask were centrifuged at 800×*g* for 10 min. Then, the supernatant was removed, and cell pellets of *Bxj*-infected RBCs were washed twice in cold cytomix buffer (120 mM KCl, 0.15 mM CaCl_2_, 10 mM K_2_HPO_4_/KH_2_PO_4_, pH 7.6, 25 mM HEPES. pH 7.6, 2 mM EGTA, 5 mM MgCl_2_, final pH 7.6) [17, 18, 22, 27–29]. Mixture, containing 1 × 10^8^ infected RBCs and 20 μg of circular *PBS-bpgb-rap-orf* plasmid in a final volume of 100 μl was transfected with parameters of 1200 V and 25 μF using a Gene Pulser Xcell™ Electroporation System (Bio-Rad Laboratories, Hercules, CA, USA). After transfection, the mixtures were transferred into wells of 24-well culture plates containing 7.5% fresh sheep RBCs and 20% FBS. After transfection for 24 h, 2 μg/ml of blasticidin-S (BSD, Gibco, R21001) was added to the cultures, and the concentration of BSD was gradually increased to 10 μg. Parasites, maintained in medium with BSD for 2–3 weeks, could be observed in thin blood smear stained with Giemsa under microscopy. To obtain a clonal strain, the population of parasites were diluted to 2.5 infected RBCs/ml with completed medium containing 7.5% of fresh sheep RBCs. Then, 100 μl of the diluted culture was added to each well of a 96-well culture plate, maintained at 37 °C in an atmosphere of 5% CO_2_ for 14–17 days. During this period, the medium was completely replaced every 3 days. Three monoclonal strains were able to grow in a high concentration of BSD (10 μg/ml) and were designated as *Bxj*-c1, *Bxj*-c2, and *Bxj*-c3.

### Identification of monoclonal parasite *Bxj* stably expressing eGFP-BSD

**Fig. 2:**
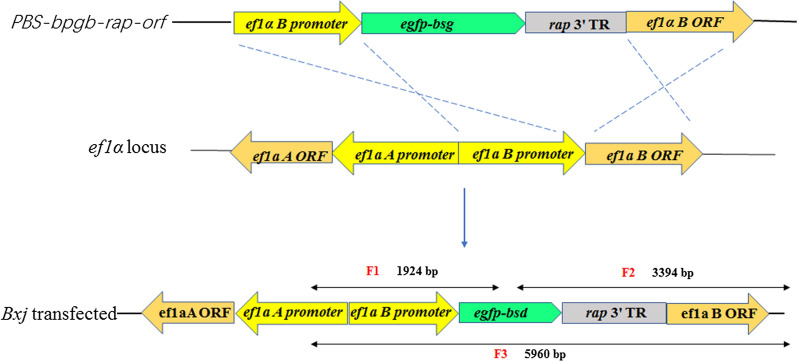
Plasmid structures and *ef1α* locus in *Bxj* genome. *PBS-bpgd-rap-orf* shows the structure of plasmid used for stable transfection in this study. *ef1α* locus illustrates the organization of *ef1α* in the *Bxj* genome. Transfected *Bxj* parasite lines, stably expressing eGFP-BSD fusion protein, should be like the *Bxj* transfected diagram

Three pairs of primer (Table 1) were designed to confirm whether the *egfp-bsd-rap* fragments were correctly integrated into the *Bxj* *ef1α* locus. The first set of primers (*efbs-F* and *efbs-R*) was designed to amplify the F1 fragment with the size of 1924 bp to determine 5′ recombination, while the second set of primers (*gfor-F* and *gfor-R*) were generated to amplify the F2 fragment of 3394 bp to confirm 3′ recombination. Additionally, a large-size fragment (F3, 5960 bp) was amplified using the third primer pair (*efbs-F* and *orf-R*) targeting the *ef1α* locus (Fig. 2).

To identify the expression of eGFP-BSD fusion protein, WB and indirect immunofluorescence assay (IFA) were performed. Briefly, when infected blood cells reached 5–10%, cultures of *Bxj*-c1, *Bxj*-c2, *Bxj*-c3, and wild-type *Bxj* were added to three times the volume of red blood cell lysis buffer (Solarbio, Beijing, China) to lyse red blood cells. Merozoites of *Bxj*-c1, *Bxj*-c2, and *Bxj*-c3 and wild-type *Bxj* (WT) were collected using centrifugation at 5000×*g* for 10 min. The merozoite pellet was mixed with a twofold volume of 1x sodium dodecyl sulfate-polyacrylamide gel electrophoresis (SDS-PAGE) loading buffer and heated at 100 °C for 5 min. Soluble proteins were subjected to SDS-PAGE and transferred to a polyvinylidene fluoride (PVDF) membrane. Then, the membrane was incubated with monoclonal anti-eGFP antibody (Sigma, SAB2702211, dilution: 1:2000) as the primary antibody followed with horseradish peroxidase (HRP)-conjugated goat anti-mouse IgG (Proteintech, USA, dilution: 1:5000), diluted with 0.1 M Tris-buffered saline (pH 7.6) with 0.1% Tween 20 (TBST). After the membranes were washed three times using TBST, bands were detected using a SuperSignal West Femto Kit (Thermo, USA) in a ChemiDoc MP imaging system (Bio-Rad, USA). IFA was performed using anti-GFP antibody (Sigma, SAB2702211, dilution: 1:200) as the primary antibody and Alexa 488-conjugated goat anti-mouse IgG (Thermo Fisher, USA, dilution: 1:500) as the secondary antibody [30]. The parasite nucleus was stained with Hoechst 33342 (Invitrogen, dilution: 1:2000) and observed with confocal laser scanning microscopy (SP8, Leica, Germany).

## Results

### An inhibitory effect of BSD on *Bxj* merozoites in in vitro culture

The inhibitory efficiency of BSD on *Bxj* merozoites in in vitro culture shows an upward trend with gradual increase of drug concentration, ranging from 1 to 10 μg/ml (Fig. 1). After 48 h of drug selection, the 50% inhibitory concentration of BSD (IC_50_) was 2.26 μg/ml. When the concentration reached 8 μg/ml, the growth of over 90% of *Bxj* merozoites was inhibited. To inhibit the growth of WT, 2–10 μg/ml BSD was employed in our following experiments.

Regarding the inhibition assays with WR99210, no efficient growth inhibition was observed. When the concentration of WR99210 increased to 150 μg/ml, the growth inhibition of *Bxj* was as low as ~ 30%. Thus, the BSD gene was selected to be included in the following constructs as a selectable marker.

### Stable transfected *Bxj*

**Fig. 3:**
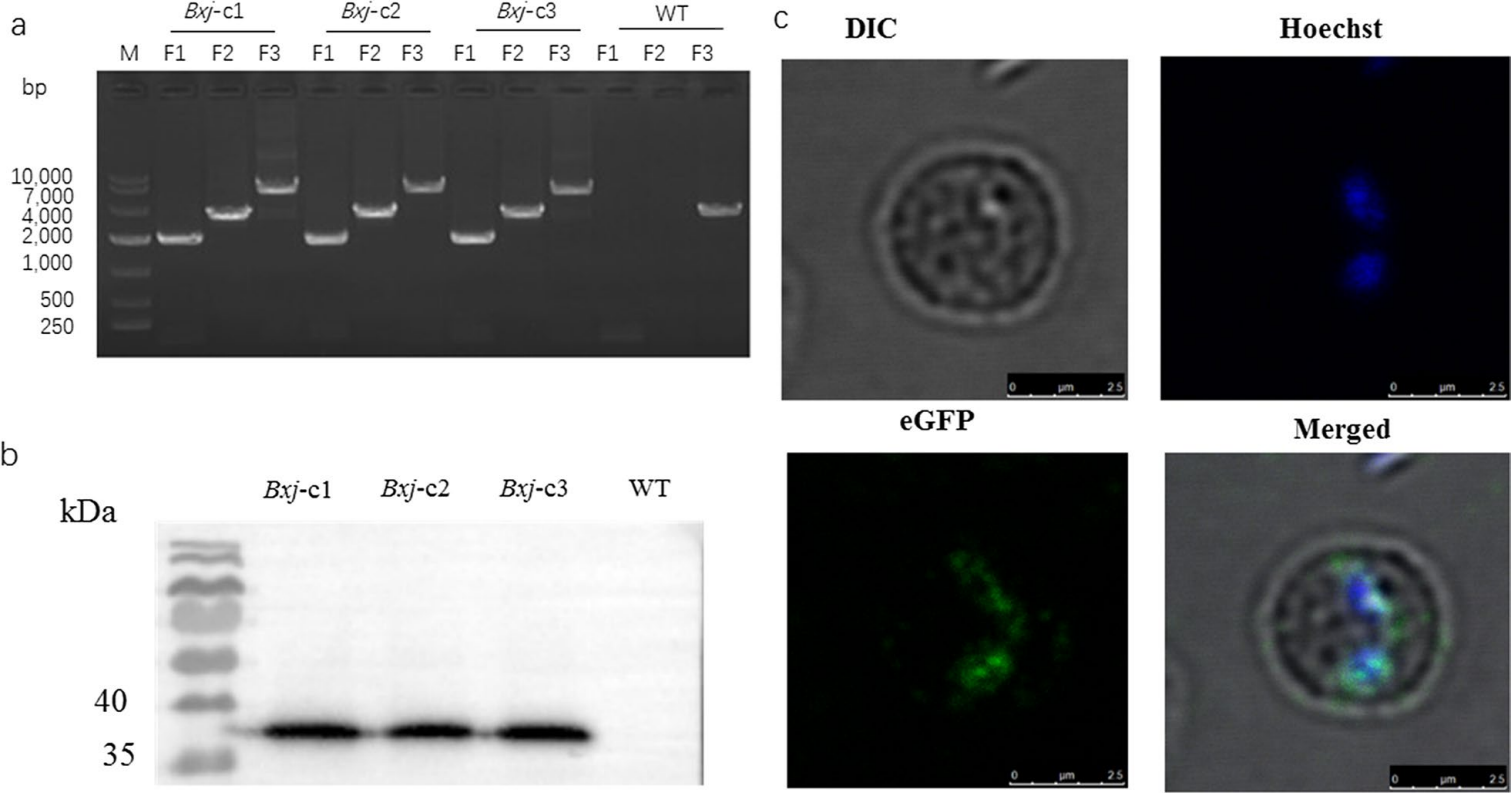
Identification of monoclonal parasite *Bxj* stably expressing eGFP-BSD. **a** PCR amplification of F1, F2, and F3 fragments. **b** Western blot analysis of stable expression of eGFP-BSD in *Bxj*-c1, *Bxj*-c2, and *Bxj*-c3. **c** IFA confirms stable eGFP expression of *Bxj*. The parasite nucleus was stained with Hoechst 33342

After 2 weeks of drug selection with BSD, transfected *Bxj* was cloned by limiting dilution. Three clonal parasite lines, designated as *Bxj*-c1, *Bxj*-c2, and *Bxj*-c3, were obtained. At this point, no fluorescence was observed in the three lines. To determine whether the fragment of *egfp-bsd-rap* was correctly integrated into the target locus, PCR amplifications were performed using three primer pairs. As show in Figs. 2 and 3a, three fragments (1924, 3394, and 5960 bp) were successfully obtained from each of the parasite lines (*Bxj*-c1, *Bxj*-c2, and *Bxj*-c3) and validated by gene sequencing. On the contrary, only one fragment (approximately 3500 bp), corresponding to the third primer pair, was amplified from WT (Fig. 3a).

### Expression analysis of eGFP-BSD fusion protein in *Bxj* merozoites

Merozoites collected from *Bxj*-c1, *Bxj*-c2, *Bxj*-c3, and WT were subjected to Western blot analysis to determine eGFP-BSD fusion protein (around 38 kDa) using monoclonal anti-eGFP. Available results showed that antibodies specifically bound to a protein of ~ 38 kDa; however, they did not react with merozoite proteins of WT (Fig. 3b). Similarly, the evidence of IFA also demonstrated expression of eGFP-BSD in *Bxj* merozoites (Fig. 3c). These three lines stably expressed eGFP for 11 months.

## Discussion

The first description of a stable transfection system in *Babesia* genus is reported in *B. bovis* in 2009; subsequently, this technique has been developed in *B. gibsoni* and *B. ovata* [17, 22, 29]. For most of public health/economically important *Babesia* spp., including *B. microti*, *B. divergens*, *B. duncani*, *B. ovis*, and *Theileria* spp., such as *T. annulata* and *T. parva*, genetic manipulation platforms have not been described*.* In addition, *Bxj* was firstly isolated from sheep infested with *R. sanguineus* and *H. anatolicum anatolicum* collected from the Xinjiang Uygur Autonomous Region in China in 2001 [31]. Prevalence of *Bxj* has been systematically investigated across China using molecular and sera techniques, such as multiplex PCR, loop-mediated isothermal amplification method, reverse line blot assay, and enzyme-linked immunosorbent assays [11, 12, 15]. Those data indicated that this pathogen was widely prevalent in sheep and goat in almost all investigated regions across China; however, genetic manipulation of *Bxj* has not been documented. In our previous study, we systematically evaluated the transient transfection parameters from *Bxj*, including transfection solution, programs, amount of plasmid DNA, and promoter activities. Eventually, a *Bxj* transient transfection system was developed with the most favorable transfection conditions (human T cell nucleoporation solution, program V-024, 20 μg of plasmid DNA, and ef1α promoter). However, an alternative transfection parameter (cytomix transfection solution, 1200 V and 25 μF, 20 μg of plasmid DNA, and ef1α promoter) also achieved preferable results.

A drug selection marker is required to develop a stable transfection system for *Bxj*. In this study, we firstly evaluated the sensitivity of *Bxj* to WR99210, which had been reported to provide suitable transfection of *Plasmodium*, *B. bovis*, and *B. gibsoni*. However, *Bxj* showed resistance to this drug. Similar situations have been described in *B. bovis* and *Plasmodium* spp. [17, 32]. This can be explained by the existence of an associated gene in the *Bxj* genome. Inhibition assays with BSD resulted in a strong *Bxj* merozoites growth inhibition with an IC_50_ of 2.26 μg/ml, validating this drug as preferable to WR99210, the selection maker. Thus, this was the selection maker used for developing *Bxj* stable transfection. Nevertheless, it is worth mentioning that the value of IC_50_ is around five times of that *B. bovis* (~ 0.4 μg/ml).

With regard to the target locus for genome integration, the *ef1a* locus was selected as the ideal target site for several *Babesia* spp., for instance *B. bovis*, *B. ovata*, and *B. gibsoni* [17, 22, 29]. Sequence analysis reveals that the *Bxj ef1α* locus consists of two identical genes, arranged head to head and separated by a 1454-bp regulator sequence. Disruption one of these two genes had no significantly negative effect on survival and growth of parasites. It is suggested that this gene locus also serves as a suitable target site to introduce foreign genes for *Bxj*. Transfected parasites could be observed under microscopy after 2 weeks of selection with BSD. PCR identification revealed that the fragment of *gfp-bsd-rap* 3' UTR was successfully introduced into the *ef1α* locus of the cloned *Bxj*-c1, *Bxj*-c2, and *Bxj*-c3. Stably expressed eGFP was confirmed by WB and IFA assays; however, green fluorescence could not be observed with fluorescence microscopy. We attempted to obtain transfected *Bxj* which could directly detect fluorescence under fluorescence microscopy, by replacing the *ef-1α* promoter with an actin promoter (approximately 2000 bp)/*ef1α*-IG A and replacing eGFP with a red fluorescent protein gene (data not shown). However, this goal was not achieved. A similar situation was previously reported in *C. parvum* [19]*.* The firefly luciferase and fluorescent proteins were not detected in transfected parasites, whereas nanoluciferase showed significant reporter activity at 48 h after transfection [19].

Although there are still some drawbacks in this system, including lack of fluorescence and relatively low transfect efficiency compared with the CRISPR/Cas9-based genome editing strategy, this transfection system of *Bxj* provides a powerful tool to determine gene function and discover critical gene families of invasion, egress, immune evasion, and even virulence factors. A more convenient, facile, and highly effective technique needs to be developed in the near future.

## Conclusions

To conclude, we provide a stable transfection system for *Bxj* and obtain transfected parasites, which have stably expressed eGFP-BSD for 11 months. This study is the first effort to create a platform for genetic manipulation of *Bxj* to further illustrate the invasive mechanism of this parasite, together with the parasite–vector and parasite–host interactions.

## Data Availability

All data are available upon request.

## Abbreviations

*Bxj*: *Babesia* sp. Xinjiang
FBS: Fetal bovine serum
RBCs: Red blood cells
PCR: Polymerase chain reaction
eGFP: Enhanced green fluorescent protein
BSD: Blasticidin
WB: Western blot
IFA: Indirect immunofluorescence assay
